# Identification of an Immune-Related Biomarker Model Based on the CircRNA-Associated Regulatory Network for Esophageal Carcinoma

**DOI:** 10.1155/2021/1334571

**Published:** 2021-11-17

**Authors:** Zhaonian Hu, Jun Xie, Xiaochun Chen, Jia Tang, Kaiguo Zhou, Song Han

**Affiliations:** ^1^College of Electronic and Information Engineering, Southwest University, Chongqing, China; ^2^Department of Cardiothoracic Surgery, The Affiliated Suzhou Science and Technology Town Hospital of Nanjing Medical University, Suzhou, Jiangsu, China

## Abstract

Esophageal carcinoma (ESCA) is one of the most frequent types of malignant tumor that has a dismal prognosis. This research applied datasets aimed from the GEO and TCGA to create a prognostic signature for forecasting the clinical outcome of ESCA patients on the basis of a circRNA-associated regulatory network. *Methods*. A regulatory network associated with ESCA was established based on transcriptome data of circRNAs, miRNAs, and mRNAs. Functional annotations were implemented to further explore the mechanism of ESCA. Cox relative regression method was applied to create a risk signature. Besides, the immune microenvironment of the signature was investigated by utilizing the CIBERSORT algorithm. *Results*. Based on 27 DEcircRNAs, 65 DEmiRNAs, and 780 DEmRNAs, the circRNA-miRNA-mRNA network was finally set up. Functional enrichment unearthed that the regulatory network might participate in phosphorylation negative regulation, MAPK pathway, and PI3K/AKT pathway. This study established a risk scoring signature based on the seven immune-related genes (IRGs) (MARP14, RASGR1, PTK2, HMGB1, DKK1, RARB, and IGF1R), which was validated for its reliability. A stable and accurate nomogram combining immune-related risk scores with clinical features was constructed. Furthermore, we observed that the risk model was also related to the immunocyte infiltration. *Conclusion*. Our study successfully created a circRNA-associated regulatory network and further developed an immune-related model to forecast the clinical outcome of ESCA patients as well as to assess their immune status.

## 1. Introduction

Esophageal cancer (ESCA) is the most frequent type of malignancy, which ranked the sixth for the mortality rate of all tumors [1]. According to GLOBOCAN 2018 data, it was estimated that around 572,000 new cases of ESCA and 508,000 fatalities occurred in that year [2], and the 5-year overall survival (OS) rate was merely 15–25% [3]. Despite improvements in the diagnosis and treatment of ESCA, most patients are not diagnosed until the middle or late stages because of the insidious nature of early symptoms. Therefore, there is an urgent need for more precise and effective targets to aid in the diagnosis and personalized therapy of ESCA patients.

CircRNAs, defined as endogenous noncoding RNAs, have a single-stranded, covalently closed-loop structure. Their closed structure is produced by reverse splicing the 5′ and 3′ ends of linear RNAs, making them more resistant to nucleic acid exonucleases, as well as more stable in tissues and plasma compared to linear RNAs [4, 5]. In 2011, the ceRNA hypothesis was first proposed, with the implication that circRNAs could negatively regulate the expression of miRNAs and influence their interactions with mRNAs [6]. In recent years, numerous studies showed the carcinogenic roles of circRNAs. CircRNA 100146, for example, could regulate SF3B3 through modulating miR-361-3p, thereby promoting the advancement of lung cancer [7]. CircNRIP1, a tumor promotor, may influence AKT1 expression in gastric cancer by binding to miR-149-5p [8]. Similarly, CircSETD3 was reported to be significantly overexpressed in nasopharyngeal carcinoma, which may dramatically enhance cancer cell invasion and migration [9]. Numerous studies indicate that certain circRNAs have cancer-suppressive effects. CircTADA2A-E6 suppresses breast cancer development by targeting miR-203a-3p [10]. Furthermore, circCUL2 has the potential to limit the progression of gastric cancer via autophagy activation mediated by miR-142-3p/ROCK2 [11].

Currently, the 8th edition of ESCA TNM staging is the primary tool used clinically to assess the prognosis of patients with ESCA [12]. However, this approach is general and limited by individual differences. Therefore, there is a pressing need to exploit new methods that can accurately evaluate the survival of ESCA. Recently, mRNA gene signatures based on immune-related genes have become a research hotspot for predicting the prognosis of patients. For example, Chen et al. developed an immune signature to forecast head and neck cancer patients' prognosis [13]. Moreover, the prognostic index model of 11 immune-related genes was constructed in hepatocellular carcinoma [14].

Although previous research has demonstrated the performance of the immune-related model in many malignancies, few have considered using immune genes to create a prognostic risk model for ESCA.

In this present study, we successfully constructed an esophageal cancer-associated ceRNA network using GEO datasets and the TCGA database. We further developed an immune-related risk signature by selecting seven immune-related genes from the ceRNA network. Finally, we further detected the expression patterns of signature genes by *in vitro* analysis. Our study not only provides an optimal prognostic model to predict ESCA patients' OS but also opens up a new insight for ESCA treatment.

## 2. Methods

### 2.1. Data Collection

The circRNA-seq data of ESCA cases (GSE131969) were collected from the GEO database (http://www.ncbi.nlm.nih.gov/geo/). The miRNA-seq data were obtained from the TCGA (https:/portal.gdc.cancer.gov/). The mRNA transcriptome data (GSE53625) and clinical data were also obtained from the GEO database. Immune-related biomarkers were extracted from ImmPort (https:/www.immport.org/).

### 2.2. Screening of Differentially Expressed RNAs

The limma package was applied to determine differentially expressed circRNAs (DEcircRNAs). Log [fold change (FC)] > 3 and adj. *p* < 0.05 were used to filter DEcircRNAs. We used the edgeR package to screen differentially expressed miRNAs (DEmiRNAs) and mRNAs (DEmRNAs), respectively. The cutoff values for DEmiRNA and DEmRNA were log [fold change (FC)] > 0.5 and adj. *p* < 0.05.

### 2.3. Development of the CircRNA-Associated Regulatory Network

The basic information of circRNAs was downloaded from circBase (http://circbase.org/). The CSCD online tool (http://gb.whu.edu.cn/CSCD/) was performed to obtain targeted miRNAs of the circRNAs, and these potential miRNAs were determined by DEmiRNAs. Next, the relationship of miRNA and mRNA was generated by the TargetScan, miRDB, and miRTarBase prediction tools [15–17]. Afterward, these target mRNAs were intersected with the determined DEmRNAs. Ultimately, a circRNA-associated regulatory network was set up by Cytoscape.

### 2.4. Functional Enrichment Analysis

Gene ontology (GO) is a bioinformatics method designed to measure gene function. KEGG, a database that integrates genomic, chemical, and biological systems' information, has been widely used to integrate and interpret datasets generated by high-throughput experimental techniques. To uncover the functional mechanism of the regulatory network, GO and KEGG methods were performed, and *p* < 0.05 was considered significant.

### 2.5. Construction of the CircRNA-Based Prognostic Signature

We first collected immune-relevant genes (IRGs) from the ImmPort database, which is a public database providing an up-to-date genes' list and functional information for IRGs. Then, the overlapping genes between IRGs and the identified mRNAs retrieved from the circRNA regulatory network were utilized to determine differentially expressed IRGs. To develop a favorable risk model, the GSE53625 set was divided into the training cohort and test cohort at 6 : 4 ratio. The prognostic IRGs were first identified by the univariate regression method. To shrink the model of overfitting, LASSO regression was conducted. Finally, multivariate Cox method was conducted to create a signature to predict OS for ESCA cases. The risk score for patients was generated by the following equation: risk score = gene 1 expression × coef 1 + gene 2 expression × coef 2… + gene *n* expression × coef *n*, where coef *n* represents the regression coefficient. The test cohort, entire cohort, and TCGA set were utilized to confirm the performance of the model.

### 2.6. Establishment of the Nomogram

To estimate the independent capacity of the prognostic model, univariate and multivariate Cox methods were carried out. A nomogram to predict OS of ESCA was developed by combining the clinical traits and prognostic model. To validate the predictive power of this signature, we plotted calibration maps and ROC curves.

### 2.7. Immunocyte Infiltration Analysis

CIBERSORT is a bioinformatics method to identify the infiltration of immune cells. In this case, we input the reference gene profiles and analyzed the infiltrating proportions of each cell type among 22 types of tumor-infiltrating immune cells (TICs).

### 2.8. Gene Set Enrichment Analysis

We applied GSEA to examine the underlying biological function related to high-risk patients in the signature. The gene sets of Hallmark and KEGG were obtained from the Molecular Signatures Database. Gene sets with a normalized *P* value less than 0.05 were deemed significant.

### 2.9. Cell Culture

Two human ESCA cell lines (KYSE-150 and Eca109), as well as one normal esophageal epithelial cell line (HEEC), were provided from the Chinese Academy of Sciences (Shanghai, China). ESCA cells and HEEC were cultured in the RPMI-1640 medium with 10% fetal bovine serum (FBS) and 1% penicillin/streptomycin with 5% CO_2_ at 37°C.

### 2.10. RNA Extraction and qRT-PCR

Total RNA was isolated from cell samples utilizing RNA-easy isolation reagent (Vazyme Biotech), which was then reversed into cDNA by PrimeScript Mix reagent (Takara). SYBR Green Premix (Vazyme Biotech) was prepared for use in the PCR system. The value of individual genes was finally standardized to the GAPDH expression level. The primer sequences for indicated genes are displayed in Supplementary Table S1.

### 2.11. Statistical Analysis

Kaplan–Meier method was employed to evaluate the clinical outcome differences between two risk groups. In addition, receiver operating characteristic (ROC) analysis was performed to examine the reliability of the model. All statistical data were analyzed using GraphPad 8.0 and R software version 4.0.

## 3. Results

### 3.1. Identification of DEcircRNAs, DEmiRNAs, and DEmRNAs

**Figure 1:**
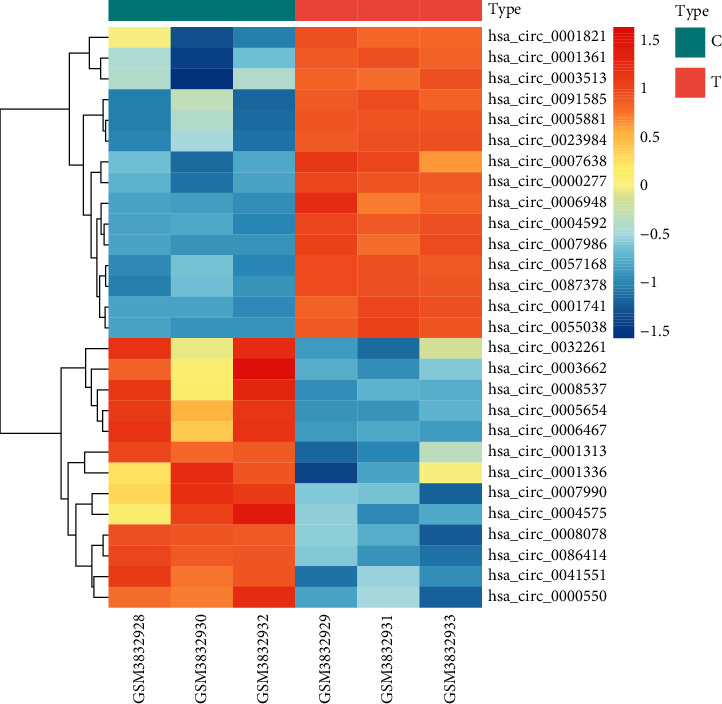
Heatmap analysis of the 28 differentially expressed circRNAs in the GSE3832930 dataset.

A total of 28 DEcircRNAs, including 15 highly expressed circRNAs and 13 lowly expressed circRNAs, were identified in the GSE131969 dataset (Figure 1). We eliminated hsa_circ_0001336 since it was not found in the CSCD database. After performing the CSCD online tool, we identified 1140 miRNAs that might target these 27 circRNAs. After crosschecking with DEmiRNAs retrieved from the TCGA database, only 65 miRNAs remained. We then predicted potential mRNAs targeted by these 65 DEmiRNAs using TargetScan, miRDB, and miRTarBase. These candidate mRNAs were intersected with DEmRNAs selected from GSE53625, resulting in 780 genes shared.

### 3.2. Construction of the ceRNA Network

**Figure 2:**
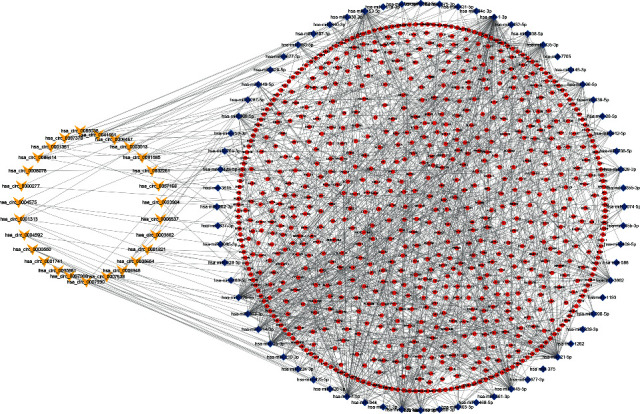
CircRNA-miRNA-mRNA ceRNA network in ESCA. V, ellipses, and diamonds represent circRNAs, miRNAs, and mRNAs, respectively.

A total of 27 DEcircRNAs, 65 DEmiRNAs, and 780 DEmRNAs were finally determined to establish a circRNA-associated network by Cytoscape 3.8 software (Figure 2).

### 3.3. Functional Enrichment Analysis

**Figure 3:**
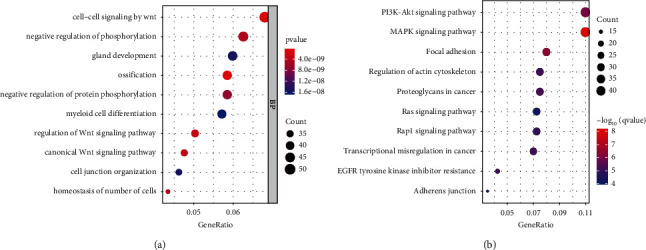
Function enrichment of mRNAs in the network. (a) Enrichment of significant GO terms. (b) Enrichment of significant KEGG pathways.

As shown in Figure 3(a), the significant function enrichments were Wnt-related pathway, negative regulation of phosphorylation, and negative regulation of protein phosphorylation. KEGG revealed that tumor pathways were greatly enriched, including the MAPK pathway, PI3K-AKT pathway, and Ras pathway (Figure 3(b)).

### 3.4. Construction of the CircRNA-Based Signature in the Training Set

**Figure 4:**
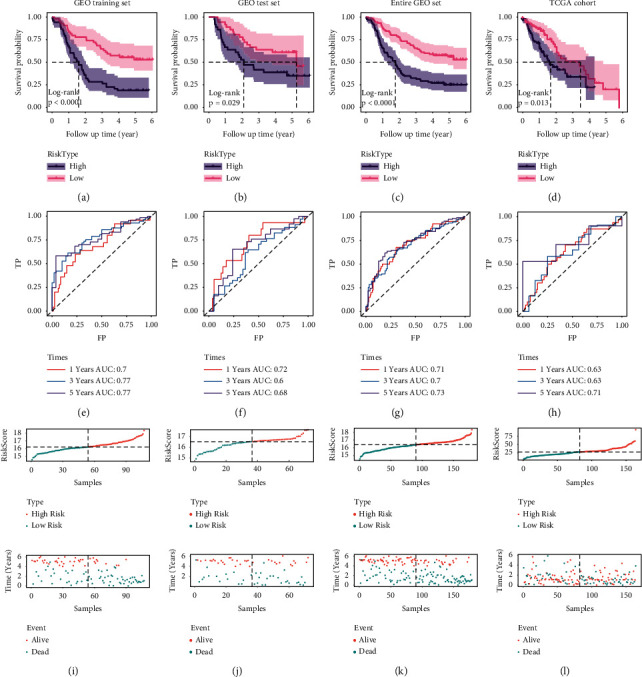
Predictive power of the seven-IRG model in ESCA. (a–d) Survival analysis for ESCA cases with low- and high-risk scores in the GEO training cohort, test cohort, entire cohort, and TCGA dataset. (e–h) ROC curve analysis of the model, 1, 3, and 5 years. (i–l) The distribution of the risk value and clinical outcome in the four cohorts.

We randomly divided GSE53625 containing 179 patients into training and internal validation series at a 6 : 4 ratio. The IRGs were overlapped with DEmRNAs from the ceRNA network mentioned above, resulting in 64 differentially expressed IRGs remained. These genes were further subjected to the univariate Cox method, LASSO Cox, and multivariate Cox analysis consecutively to establish a seven-IRG signature (Table 1). The risk score = 0.745 × HMGB1 + 0.363 × PTK2 + 0.261 × IGF1R + 0.228 × RARB + 0.149 × DKK1 – 0.192 × RASGRP1 – 0.353 × MAPK14. As expected, the clinical outcome of the high-risk group was dramatically dismal than that of the low-risk group (Figure 4(a)). The prognosis accuracy of our model was assessed by the areas under the curve (AUC) of the ROC (Figure 4(e)). In addition, we explored the distribution of the risk value and survival outcome of each patient (Figure 4(i)).

### 3.5. Validation of the CircRNA-Based Signature

Our proposed signature is verified to be efficient when tested in the internal cohort. We observed that high-risk ESCA patients showed worse OS than its counterpart (Figure 4(b)). The similar result was also confirmed in the entire set and TCGA cohort (Figure 4(c)). The AUC of ROC was calculated to estimate the reliability of the model in the above cohorts (Figures 4(f)–4(h)). At final, the distribution of the risk score and clinical outcome of each ESCA case are shown in Figures 4(j)–4(l)).

### 3.6. Establishment of the Nomogram Based on the Risk Model

**Figure 5:**
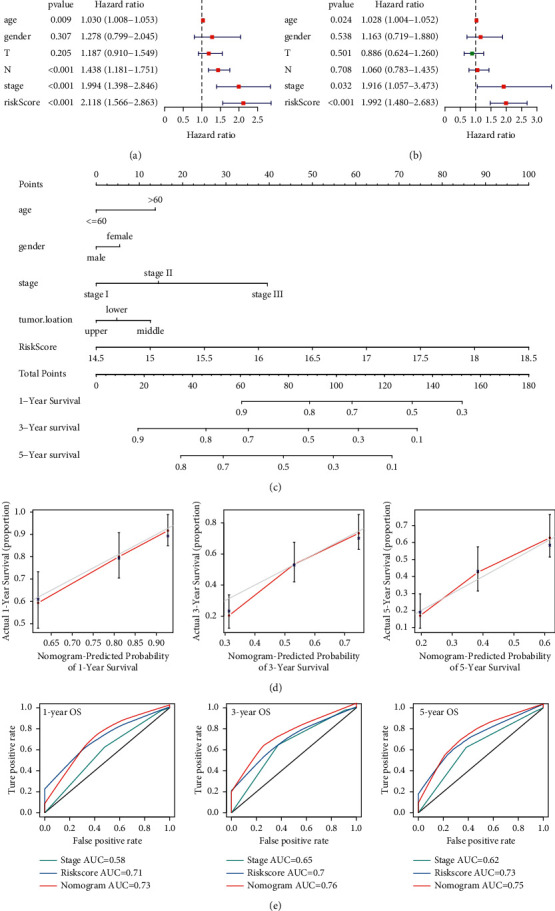
Cox regression analyses and a nomogram of the seven-IRG model in ESCA. (a-b) Forest diagrams of univariate and multivariate Cox regressions of the risk score and clinical parameters. (c) A nomogram for forecasting the clinical outcome of ESCA cases. (d-e) Calibration and ROC curves are employed to confirm the predictive ability of the nomogram.

To evaluate the independent power of the prognostic signature, Cox relative hazard regression methods were conducted (Figures 5(a) and 5(b)). We found that only the risk score was greatly meaningful for forecasting OS in univariate and multivariate Cox methods. In addition, a nomogram was developed by combining the risk model and clinical parameters for forecasting the clinical outcome of ESCA cases (Figure 5(c)). Calibration curves showed excellent consistency between predictive and actual survival rates (Figure 5(d)). The AUC values achieved 0.73, 0.76, and 0.75 for 1, 3, and 5 years, respectively (Figure 5(e)).

### 3.7. Correlation Analysis of Immunocyte Infiltration and Risk Score

**Figure 6:**
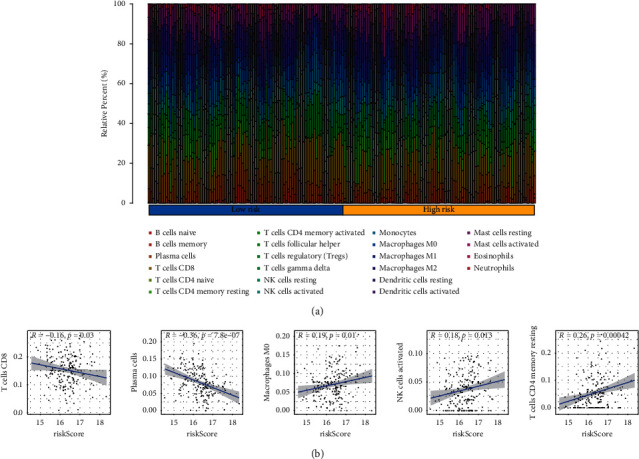
Immune landscape of ESCA patients. (a) The proportion of infiltrative immune cells in two risk groups. (b) The correlation of the risk score with CD8^+^ T cells, plasma cells, M0 macrophages, activated NK cells, and resting memory CD4^+^ T cells.

By applying the CIBERSORT algorithm, a bar plot (Figure 6(a)) was performed to represent the discrepancy in the infiltration level of 22 types of TICs between two risk groups. To further confirm the relationship of the risk signature and immunocyte infiltration in ESCA, the Pearson correlation analysis was performed. The result showed that M0 macrophage, resting memory CD4^+^ T cell, and activated NK cells had a positive association with the risk score, while CD8^+^ T cells and plasma cells displayed a negative correlation with the risk score (Figure 6(b)).

### 3.8. Prognostic Performance of the CircRNA-Based Signature in the Immunosuppressive Microenvironment

**Figure 7:**
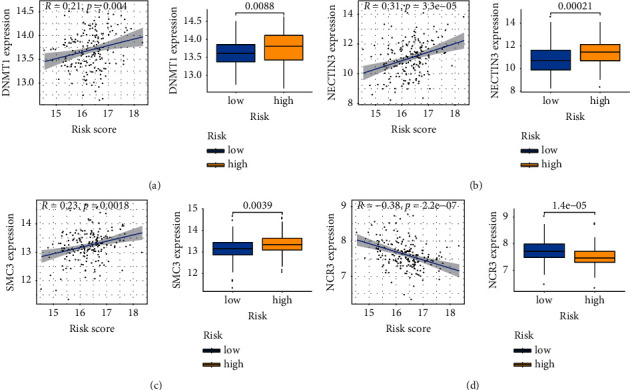
Association between the signature and the negative regulatory biomarkers related to the cancer-immunity cycle. (a) DNMT1. (b) NECTIN3. (c) SMC3. (d) NCR3.

Recently, the cancer-immunity cycle, which depicts the immune surveillance on tumor cells, has become a research highlight in cancer immunotherapy. It involves a series of procedures including cancer cell antigen release [18]. We selected 54 negative regulatory genes involved in the cancer-immunity cycle from the Tracking Tumor Immunophenotype website for further analysis. Next, we analyzed the relationship between negative regulatory genes and our signature. The results suggested that DNMT1, NECTIN3, and SMC3 had a positive correlation with the risk score, while NCR3 presented the opposite trend (Figure 7).

### 3.9. GSEA

**Figure 8:**
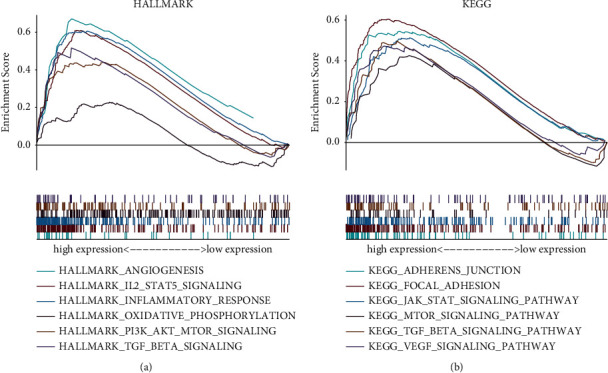
GSEA. (a) Hallmark gene set. (b) KEGG gene set.

As shown in Figure 8(a), angiogenesis, IL2/STAT5 pathway, inflammatory response, oxidative phosphorylation, PI3K/AKT/MTOR pathway, and TGF-beta pathway were dramatically enriched in cases with high-risk scores. We also observed that six KEGG pathways were activated in the high-risk group, including the MTOR pathway and TGF-beta pathway, which are in line with the result of Hallmark (Figure 8(b)).

### 3.10. Detection of Signature Gene Expressions in Cell Lines by the qRT-PCR Assay

**Figure 9:**
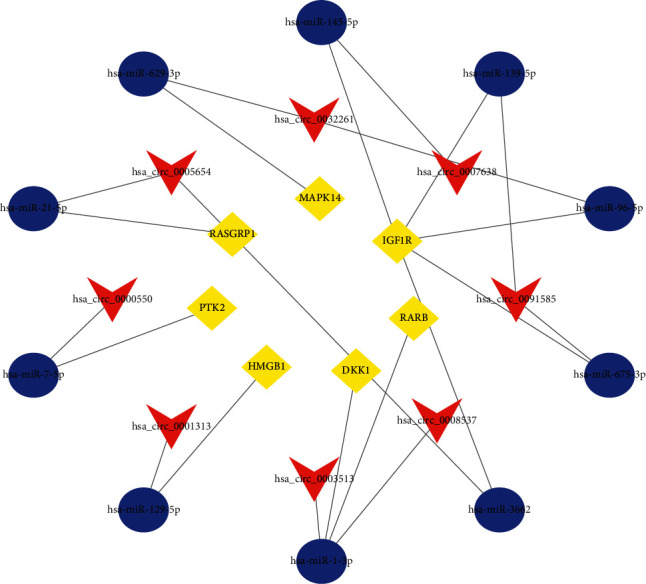
Construction of the signature-based ceRNA subnetwork based on the seven-IRG model. V, ellipses, and diamonds represent circRNAs, miRNAs, and mRNAs, respectively.

**Figure 10:**
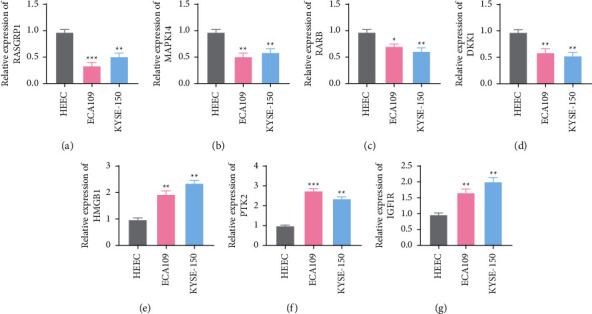
Expression of seven model genes in ESCA cell lines and human normal esophageal epithelial cell lines. (a) RASGRP1. (b) MAPK14. (c) RARB. (d) DKK1. (e) HMGB1. (f) PTK2. (g) IGF1R (^*∗*^*p* < 0.05; ^*∗∗*^*p* < 0.01; ^*∗∗∗*^*p* < 0.001).

Finally, a circular RNA-associated ceRNA subnetwork was constructed based on our immune-related risk signature (Figure 9). Subsequently, we further examined the expression levels of seven signature genes in HEEC, Eca109, and KYSE-150 using qRT-PCR. The result showed that HMGB1, PTK2, and IGF1R had significantly higher expression levels in Eca109 and KYSE-150 and lower expression levels in HEEC. On the contrary, RASGRP1, MAPK14, RARB, and DKK1 were lowly expressed in Eca109 and KYSE-150 and highly expressed in HEEC (Figure 10).

## 4. Discussion

ESCA ranks eighth and sixth among cancers according to morbidity and mortality, respectively, with high rates of metastasis and recurrence [1]. Consequently, it is urgent to exploit powerful biomarkers for ESCA. Accumulating evidence suggests that circRNAs could interact with miRNA, resulting in a corresponding decrease in miRNA activity [19]. miRNAs act primarily on mRNAs, but the circRNA competition process impedes the regulation of target gene expression by miRNAs, which further affects tumor formation, development, and metastasis. For example, hsa_circ_0006168 could boost ESCC cell viability and metastasis by binding with miR-100 [20]. As discovered by Meng et al., CIRS-7 could accelerate the development of ESCC by binding with miR-876-5p and upregulating the MAGE-A family gene [21]. In addition, circGSK3*β* is reported to act as an indicator for the prognosis of ESCA [22]. Thus, circRNA may become a new molecular target for ESCA treatment.

We identified 27 DEcircRNAs, 65 DEmiRNAs, and 780 DEmRNAs from GEO, TCGA, and CSCD databases, which in turn establish an ESCA-associated ceRNA network. According to the immune-related signature, we also created a circRNA-miRNA-mRNA subnetwork. Wang and his colleagues proved that hsa_circ_0005654 had higher expression in early gastric carcinoma specimens than in normal cases [23]. hsa_circ_0001313, also named circCCDC66, has been shown to have a cancer-promoting role in stomach cancer, colon cancer, kidney carcinoma, and lung cancer [24–27]. However, other six circRNAs have not been studied. Hence, the role of these circRNAs in tumors, especially in ESCA, needs to be unearthed in future research studies.

To uncover the underlying mechanism of the ceRNA network, we implemented a functional annotation of mRNA from the network. The results showed that the Wnt-related pathway, negative regulation of phosphorylation, and negative regulation of protein phosphorylation were greatly enriched. Enrichment of the KEGG signaling pathway suggested that tumor-related pathways were activated including the MAPK pathway, PI3K-AKT pathway, and Ras pathway. MAPK signaling pathway has the basic function of regulating cell growth, survival, and differentiation. As one of the clearest signaling pathways in tumor biology research, its oncogenic effect when abnormally activated has been demonstrated in many tumors, including esophageal cancer [28]. The mechanisms by which the PI3K-AKT signaling pathway facilitates cancer development may include the following: cell proliferation, metabolic reprogramming, suppressing autophagy, and promoting EMT [29]. Currently, PI3K is promising as a new therapeutic option in lung cancer, head and neck carcinoma, and breast cancer [30–32]. Similarly, silencing G3BP1 blocks the development of ESCA via the Wnt/*β*-catenin pathway [33]. As revealed by Wang et al., SOX9 could repress the PI3K-AKT pathway in ESCC through blocking miR-203a transcription [34]. Additionally, miR302a inhibits ESCA cell viability via the MAPK signaling pathway [35].

In the present study, Cox relative regressions and LASSO regression were performed to establish a signature of seven IRGs, including HMGB1, PTK2, RASGRP1, MAPK14, IGF1R, RARB, and DKK1. HMGB1, a chromatin-associated protein, has been reported to regulate ESCC cell proliferation and radiosensitivity [36]. Di and his colleagues observed that overexpression of HMGB1 could promote cell radioresistance after irradiation. PTK2, also known as FAK, was found to facilitate metastasis of ESCC cells through the miR-4324/FAK pathway [37]. As suggested by Ma et al., high IGF1R expressions mediate cell proliferation and apoptosis, indicating that it could act as a crucial oncogene in esophageal cancer [38]. Moreover, Lyros et al. discovered that the upregulation of DKK1 in esophageal adenocarcinoma enhances tumor growth and progression through AKT-phosphorylation and the Wnt axis [39].

Recently, immune cells have been proved to play a central part in the tumor environment [40, 41]. Moreover, infiltration of immune cells has been characterized as a reliable prognostic factor in esophageal cancer. Previous reports suggested that high infiltration levels of M2 macrophages [42, 43] and Tregs [44] were strong evidence for tumor development and metastasis. Conversely, Schumacher et al. revealed that increased enrichment of CD8^+^ T cells was a favorable prognostic indicator for the clinical outcome [45]. We observed that CD8^+^ T cells are negatively associated with risk scores, consistent with the hypothesis that a high abundance of CD8^+^ T cells may indicate a better clinical outcome. In a word, our study highlights the importance of maintaining certain immune cells in tumor immunotherapy. Moreover, we found that the risk score was also related to the expression of CIC negative regulatory markers. DNMT1 belonged to the DNA methyltransferase family, and its overexpression is identified in human T-cell, B-cell, and myeloid malignancies, indicating that DNMT1 may play a crucial role in tumor maintenance [46, 47]. Nectin-3, a nectin family member, took part in regulating the formation of adherent junctions and had been reported to be a novel biomarker in tumorigenesis. It has also been demonstrated that Nectin-3 could contribute to lymphocyte extravasation by interacting with Nectin-2 [48]. The function of SMC3 (structural maintenance of chromosomes 3) was described to induce tumorigenesis, and its expression can be downregulated in CD8^+^ T cells after immunotherapy [49].

However, this study still had some limitations. Firstly, the construction of the ceRNA network was mainly based on bioinformatics analysis, and its regulatory mechanism required further experiments. In addition, the result of the correlation analysis between the signature and immunocyte infiltration and CIC negative regulatory genes needs to be validated in additional cohorts.

## 5. Conclusion

This study determined a range of DEcircRNAs, DEmiRNAs, and DEmRNAs and set up an ESCA-associated network in ESCA. Furthermore, we successfully developed an immune-related prognostic model which could accurately forecast clinical outcomes, mirror the immune microenvironment, and provide individualized treatment for ESCA patients.

## Figures and Tables

**Table 1:** Seven immune-related prognostic mRNAs significantly associated with the clinical outcome.

Gene	Coefficient	Hazard ratio (95% CI)	*p* value
HMGB1	0.745	2.11 (0.84–5.3)	0.113
PTK2	0.363	1.44 (0.83–2.5)	0.198
IGF1R	0.261	1.30 (0.81–2.1)	0.277
RARB	0.228	1.26 (1.02–1.6)	0.033
DKK1	0.149	1.16 (1.00–1.3)	0.046
RASGRP1	−0.192	0.83 (0.63–1.1)	0.175
MAPK14	−0.353	0.70 (0.36–1.4)	0.298

## Data Availability

All datasets generated for this study can be found in The Cancer Genome Atlas (https://portal.gdc.cancer.gov/) and the NCBI Gene Expression Omnibus (GSE131969 and GSE53625).

## Supplementary Materials

Supplementary Materials Supplementary Table 1: primers utilized for qRT-PCR.

## References

[1] Arnal M. J. D., Ferrandez Arenas A., Lanas Arbeloa A. (2015). Esophageal cancer: risk factors, screening and endoscopic treatment in Western and Eastern countries. *World Journal of Gastroenterology*.

[2] Bray F., Ferlay J., Soerjomataram I., Siegel R. L., Torre L. A., Jemal A. (2018). Global cancer statistics 2018: GLOBOCAN estimates of incidence and mortality worldwide for 36 cancers in 185 countries. *CA: A Cancer Journal for Clinicians*.

[3] Pennathur A., Gibson M. K., Jobe B. A., Luketich J. D. (2013). Oesophageal carcinoma. *The Lancet*.

[4] Chen L.-L. (2020). The expanding regulatory mechanisms and cellular functions of circular RNAs. *Nature Reviews Molecular Cell Biology*.

[5] Memczak S., Jens M., Elefsinioti A. (2013). Circular RNAs are a large class of animal RNAs with regulatory potency. *Nature*.

[6] Salmena L., Poliseno L., Tay Y., Kats L., Pandolfi P. P. (2011). A ceRNA hypothesis: the Rosetta Stone of a hidden RNA language?. *Cell*.

[7] Chen L., Nan A., Zhang N. (2019). Circular RNA 100146 functions as an oncogene through direct binding to miR-361-3p and miR-615-5p in non-small cell lung cancer. *Molecular Cancer*.

[8] Zhang X., Wang S., Wang H. (2019). Circular RNA circNRIP1 acts as a microRNA-149-5p sponge to promote gastric cancer progression via the AKT1/mTOR pathway. *Molecular Cancer*.

[9] Tang L., Xiong W., Zhang L. circSETD3 regulates MAPRE1 through miR-615-5p and miR-1538 sponges to promot.

[10] Xu J.-Z., Shao C.-C., Wang X.-J. (2019). circTADA2As suppress breast cancer progression and metastasis via targeting miR-203a-3p/SOCS3 axis. *Cell Death & Disease*.

[11] Peng L., Sang H., Wei S. (2020). circCUL2 regulates gastric cancer malignant transformation and cisplatin resistance by modulating autophagy activation via miR-142-3p/ROCK2. *Molecular Cancer*.

[12] Rice T. W., Ishwaran H., Ferguson M. K., Blackstone E. H., Goldstraw P. (2017). Cancer of the esophagus and Esophagogastric junction: an eighth edition staging primer. *Journal of Thoracic Oncology*.

[13] Chen Y., Li Z.-Y., Zhou G.-Q., Sun Y. (2020). An immune-related gene prognostic index for head and neck squamous cell carcinoma. *Clinical Cancer Research*.

[14] Dai Y., Qiang W., Lin K., Gui Y., Lan X., Wang D. (2020). An immune-related gene signature for predicting survival and immunotherapy efficacy in hepatocellular carcinoma. *Cancer Immunology, Immunotherapy*.

[15] Wong N., Wang X. (2015). miRDB: an online resource for microRNA target prediction and functional annotations. *Nucleic Acids Research*.

[16] Chou C.-H., Shrestha S., Yang C.-D. (2018). miRTarBase update 2018: a resource for experimentally validated microRNA-target interactions. *Nucleic Acids Research*.

[17] Fromm B., Billipp T., Peck L. E. (2015). A uniform system for the annotation of vertebrate microRNA genes and the evolution of the human microRNAome. *Annual Review of Genetics*.

[18] Chen D. S., Mellman I. (2013). Oncology meets immunology: the cancer-immunity cycle. *Immunity*.

[19] Zhong Y., Du Y., Yang X. (2018). Circular RNAs function as ceRNAs to regulate and control human cancer progression. *Molecular Cancer*.

[20] Shi Y., Guo Z., Fang N. (2019). hsa_circ_0006168 sponges miR-100 and regulates mTOR to promote the proliferation, migration and invasion of esophageal squamous cell carcinoma. *Biomedicine & Pharmacotherapy*.

[21] Sang M., Meng L., Sang Y. (2018). Circular RNA ciRS-7 accelerates ESCC progression through acting as a miR-876-5p sponge to enhance MAGE-A family expression. *Cancer Letters*.

[22] Hu X., Wu D., He X. (2019). circGSK3*β* promotes metastasis in esophageal squamous cell carcinoma by augmenting *β*-catenin signaling. *Molecular Cancer*.

[23] Wang Y., Xu S., Chen Y., Zheng X., Li T., Guo J. (2019). Identification of hsa_circ_0005654 as a new early biomarker of gastric cancer. *Cancer Biomarkers*.

[24] Xu G., Chen Y., Fu M. (2020). Circular RNA CCDC66 promotes gastric cancer progression by regulating c-Myc and TGF-*β* signaling pathways. *Journal of Cancer*.

[25] Yang J., Yang L., Li S., Hu N. (2020). HGF/c-Met promote renal carcinoma cancer stem cells enrichment through upregulation of cir-CCDC66. *Technology in Cancer Research and Treatment*.

[26] Feng J., Li Z., Li L., Xie H., Lu Q., He X. (2020). Hypoxia‑induced circCCDC66 promotes the tumorigenesis of colorectal cancer via the miR‑3140/autophagy pathway. *International Journal of Molecular Medicine*.

[27] Wang Y., Zhao W., Zhang S. (2020). STAT3-induced upregulation of circCCDC66 facilitates the progression of non-small cell lung cancer by targeting miR-33a-5p/KPNA4 axis. *Biomedicine & Pharmacotherapy*.

[28] Wang C., Li Z., Shao F. (2017). High expression of Collagen Triple Helix Repeat Containing 1 (CTHRC1) facilitates progression of oesophageal squamous cell carcinoma through MAPK/MEK/ERK/FRA-1 activation. *Journal of Experimental & Clinical Cancer Research*.

[29] Aoki M., Fujishita T. (2017). Oncogenic roles of the PI3K/AKT/mTOR Axis. *Current Topics in Microbiology and Immunology*.

[30] Tan A. C. (2020). Targeting the PI3K/Akt/mTOR pathway in non‐small cell lung cancer (NSCLC). *Thoracic Cancer*.

[31] Ciruelos Gil E. M. (2014). Targeting the PI3K/AKT/mTOR pathway in estrogen receptor-positive breast cancer. *Cancer Treatment Reviews*.

[32] Marquard F. E., Jücker M. (2020). PI3K/AKT/mTOR signaling as a molecular target in head and neck cancer. *Biochemical Pharmacology*.

[33] Zhang L. N., Zhao L., Yan X. L., Huang Y. H. (2019). Loss of G3BP1 suppresses proliferation, migration, and invasion of esophageal cancer cells via Wnt/*β*‐catenin and PI3K/AKT signaling pathways. *Journal of Cellular Physiology*.

[34] Wang L., Zhang Z., Yu X. (2020). SOX9/miR-203a axis drives PI3K/AKT signaling to promote esophageal cancer progression. *Cancer Letters*.

[35] Xia D., Tian S., Chen Z., Qin W., Liu Q. (2018). miR302a inhibits the proliferation of esophageal cancer cells through the MAPK and PI3K/Akt signaling pathways. *Oncology Letters*.

[36] Di X., He G., Chen H. (2019). High‐mobility group box 1 protein modulated proliferation and radioresistance in esophageal squamous cell carcinoma. *Journal of Gastroenterology and Hepatology*.

[37] Zhou J., Zhu J., Jiang G., Feng J., Wang Q. (2019). Downregulation of microRNA-4324 promotes the EMT of esophageal squamous-cell carcinoma cells via upregulating FAK. *OncoTargets and Therapy*.

[38] Ma W., Zhang T., Pan J. (2014). Assessment of insulin-like growth factor 1 receptor as an oncogene in esophageal squamous cell carcinoma and its potential implication in chemotherapy. *Oncology Reports*.

[39] Lyros O., Lamprecht A. K., Nie L. (2019). Dickkopf-1 (DKK1) promotes tumor growth via Akt-phosphorylation and independently of Wnt-axis in Barrett’s associated esophageal adenocarcinoma. *American journal of cancer research*.

[40] Gajewski T. F., Schreiber H., Fu Y.-X. (2013). Innate and adaptive immune cells in the tumor microenvironment. *Nature Immunology*.

[41] Lei X., Lei Y., Li J.-K. (2020). Immune cells within the tumor microenvironment: biological functions and roles in cancer immunotherapy. *Cancer Letters*.

[42] Yagi T., Baba Y., Okadome K. (2019). Tumour-associated macrophages are associated with poor prognosis and programmed death ligand 1 expression in oesophageal cancer. *European Journal of Cancer*.

[43] Yamamoto K., Makino T., Sato E. (2020). Tumor‐infiltrating M2 macrophage in pretreatment biopsy sample predicts response to chemotherapy and survival in esophageal cancer. *Cancer Science*.

[44] Yue Y., Lian J., Wang T. (2020). Interleukin‐33‐nuclear factor‐*κ*B‐CCL2 signaling pathway promotes progression of esophageal squamous cell carcinoma by directing regulatory T cells. *Cancer Science*.

[45] Schumacher K., Haensch W., Roefzaad C., Schlag P. M. (2001). Prognostic significance of activated CD8(+) T cell infiltrations within esophageal carcinomas. *Cancer Research*.

[46] Ramaswamy S., Tamayo P., Rifkin R. (2001). Multiclass cancer diagnosis using tumor gene expression signatures. *Proceedings of the National Academy of Sciences*.

[47] Andersson A., Ritz C., Lindgren D. (2007). Microarray-based classification of a consecutive series of 121 childhood acute leukemias: prediction of leukemic and genetic subtype as well as of minimal residual disease status. *Leukemia*.

[48] Devilard E., Xerri L., Dubreuil P., Lopez M., Reymond N. (2013). Nectin-3 (CD113) interacts with Nectin-2 (CD112) to promote lymphocyte transendothelial migration. *PLoS One*.

[49] Vormehr M., Reinhard K., Blatnik R. (2019). A non-functional neoepitope specific CD8+ T-cell response induced by tumor derived antigen exposure in vivo. *OncoImmunology*.

