# Supplementary material for: Identification of an Immune-Related Biomarker Model Based on the CircRNA-Associated Regulatory Network for Esophageal Carcinoma
Source: J Oncol. 2021 Nov 17;2021:1334571. doi: 10.1155/2021/1334571 (PMC8612787; doi:10.1155/2021/1334571)
Supplement: Supplementary Materials — Supplementary Table 1: primers utilized for qRT-PCR. [file 1334571.f1.pdf]

Supplementary Table 1. Primers utilized for qRT-PCR

Gene	5'to 3'
RASGRP1	Forward ACATCACCCAGTTCCGAATGA<br>Reverse GCTGTCAATGAGATCGTCCAG
MAPK14	Forward TCAGTCCATCATTCATGCGAAA<br>Reverse AACGTCCAACAGACCAATCAC
RARB	Forward CCCCAGAACAAGACACCATGA<br>Reverse TTTTGTCGGTTCCTCAAGGTC
DKK1	Forward CCTTGAACTCGGTTCTCAATTCC<br>Reverse CAATGGTCTGGTACTTATTCCCG
HMGB1	Forward TATGGCAAAAGCGGACAAGG<br>Reverse CTTCGCAACATCACCAATGGA
PTK2	Forward AGTGGACCAGGAAATTGCTTTG<br>Reverse GTGTTTTGGCCTTGACAGAATC
IGF1R	Forward AGGATATTGGGCTTTACAACCTG<br>Reverse GAGGTAACAGAGGTCAGCATTTT
GAPDH	Forward GGAGCGAGATCCCTCCAAAAT<br>Reverse GGCTGTTGTCATACTTCTCATGG
